# Impact of Micro-RNAs as biomarkers for end-stage renal disease related to hypertension and diabetes

**DOI:** 10.1038/s41598-025-16013-5

**Published:** 2025-08-24

**Authors:** Ali Ata, Doaa Ezzat, Hoda S. Sherkawy, Amr E. Ahmed, Mie Afify, Mohamed D.E. Abdelmaksoud, Samir Azazy, Weaam Gouda

**Affiliations:** 1 Department of Biotechnology and Life Sciences, Faculty of Postgraduate Studies for Advanced Sciences, Beni-Suef University, Beni-Suef, Egypt; 2 Medical Biochemistry and Molecular Biology Department, College of Oral and Dental Surgery, Misr University for Science and technology, Giza, Egypt; 3 Medical Biochemistry Department, Faculty of Medicine, Aswan University, Aswan, Egypt; 4 Biochemistry Department, Biotechnology Research Institute, National Research Centre, Giza, Egypt; 5 Surgical urology Department, Ain Shams University, Cairo, Egypt

**Keywords:** End-stage renal disease (ESRD), Hypertension (HTN), Diabetes mellitus (DM), MicroRNAs (miRNAs), Biochemistry, Biotechnology, Molecular biology, Biomarkers, Diseases, Health care, Medical research

## Abstract

End-stage renal disease (ESRD) is a rapidly increasing global health and healthcare challenge. MicroRNAs (miRNAs) have been implicated in kidney disease due to their role in apoptosis, cell proliferation, differentiation, and development. The aim of this study was to determine the role of miRNA-21-5p, miRNA-126-3p, and miRNA-192-5p in the prognosis of ESRD. In addition, we aimed to evaluate the discriminatory ability of these miRNAs as biomarkers for ESRD in relation to the comorbidities of hypertension (HTN) and diabetes mellitus (DM). One hundred and ten individuals were recruited for our study and divided into three groups: group 1 included 40 ESRD patients with hypertension, group 2 included 40 ESRD patients with diabetes, and group 3 (*n* = 30) served as healthy controls. Real-time polymerase chain reaction (RT-PCR) was used to quantify the above miRNAs. Patients with ESRD were found to have lower levels of miRNA-126-3p and higher levels of miRNAs-21-5p and − 192-5p. Furthermore, the accuracies of ROC analyses for miR-21-5p, miR-126-3p, and miR-192-5p were 96.65%, 99.5%, and 93.35% in ESRD with HTN and 95%, 71.5%, and 93% in ESRD with DM. Dysregulation of these miRNAs is associated with the development of ESRD and could be used as biomarkers for ESRD. This study briefly outlines the challenges associated with miRNA research and the potential use of miRNA molecules in the management of ESRD, proposing a research approach emphasizing the development of standardized and reliable biomarkers for therapeutic use. Despite the promising diagnostic utility demonstrated, the lack of cross-validation and external validation remains an important limitation. Future large-scale, independent studies are essential to confirm these findings and ensure broader applicability.

## Introduction

Chronic kidney disease (CKD) is a progressive condition characterized by the gradual decline in renal function, leading to significant morbidity and mortality worldwide^1^. The increasing prevalence of CKD is primarily driven by the rise in hypertension (HTN) and diabetes mellitus (DM), two of the most prevalent risk factors for renal impairment^2^. In its early stages, CKD is often asymptomatic, leading to delayed diagnosis until the disease has advanced significantly^3^. When left untreated, CKD can progress to end-stage renal disease (ESRD), a severe and often irreversible state of kidney failure^4^. Persistent renal injury over time results in structural changes such as hypoxia, tubular atrophy, and capillary rarefaction, all of which contribute to a decline in filtration capacity and eventual ESRD^5^. The growing incidence of ESRD presents a major healthcare challenge, particularly in low- and middle-income countries, where limited access to treatment disproportionately affects vulnerable populations. The epidemiological patterns of ESRD are shaped by genetic, environmental, and socioeconomic factors that vary widely across regions^6^.

MicroRNAs (miRNAs) are small, non-coding RNA molecules, typically 18–25 nucleotides in length that are detectable in various forms in blood. These molecules regulate gene expression by binding to the 3′-untranslated regions of specific messenger RNAs, thereby modulating protein synthesis^7^. Notably, miRNAs are remarkably stable in the bloodstream, making them promising candidates as diagnostic and prognostic biomarkers for various diseases^8–11^. Aberrant miRNA expression has been implicated in the pathogenesis and progression of several conditions, including diabetes, cardiovascular disorders, and kidney diseases^1,12–14^. In the context of CKD, accumulating evidence suggests that specific miRNAs may play critical roles in renal pathology, as observed in cellular and animal studies as well as in limited human research^1^. Despite growing interest, data regarding the clinical relevance of miRNA levels in CKD remain sparse, underscoring the need for further investigation^15–17^.

Although several studies have implicated miR-21, miR-126-3p, and miR-192 in the development and progression of chronic kidney disease, the findings remain inconsistent and vary across patient populations^1,14,18^. Moreover, data regarding the expression patterns of these circulating miRNAs in patients with both HTN and DM who have progressed to end-stage renal disease are notably sparse. Hence, the purpose of this study was to assess the circulating levels of miR-21, miR-126-3p, and miR-192 in ESRD patients with concurrent hypertension and diabetes and to evaluate their potential as blood-based biomarkers for identifying ESRD in this high-risk group.

## Subjects and methods

### Subjects

This study involved a cohort of 80 end-stage renal disease patients recruited from the Urology Department at El-Kasr El-Ainy Hospital, Cairo University, Egypt, along with 30 age- and sex-matched healthy control subjects. All patients met the criteria for ESRD stage 5 (G5), characterized by a glomerular filtration rate (GFR) below 15 ml/min per 1.73 m² and the need for dialysis^19^. Participants were stratified into three distinct groups: group 1 included 40 ESRD patients with HTN, defined according to the European Society of Hypertension guidelines^20^ and they had no prior history for DM; group 2 included 40 ESRD (G5) patients diagnosed with DM based on the diagnostic criteria established by the American Diabetes Association^21^ and they had no history of HTN; and group 3, the control group, included 30 healthy participants with no history of diabetes, hypertension, kidney, or cardiovascular diseases. These controls were confirmed to have normal blood pressure and blood glucose levels and were free of chronic systemic conditions. The treatment status for diabetes mellitus, hypertension, and end-stage renal disease was recorded at the time of sample collection, and all participants continued their prescribed standard treatments for these conditions. Blood samples were drawn from patients when they were admitted to the hospital and before they were given any further treatment. Individuals with hematological disorders, acute or chronic infections, significant liver dysfunction, or malignancies were excluded from participation. The study protocol received ethical approval (no. 13060121-3) from the Medical Ethics Committee of the National Research Centre, Egypt, in accordance with the Declaration of Helsinki. Informed consent was obtained from all participants prior to enrollment.

## Samples collection and analyses

Venous blood samples (5 mL) were collected from all participants. Serum was obtained by centrifugation and used for both routine biochemical analyses and miRNA profiling. Additionally, whole blood was collected in EDTA tubes for the assessment of glycated hemoglobin (HbA1c). For miRNA extraction from serum, the RNeasy Mini Kit (Qiagen Inc., Germantown, MD, USA) was employed according to the manufacturer’s protocol. The isolated miRNAs were then reverse transcribed into complementary DNA (cDNA) using the RT kit (Applied Biosystems, Foster City, CA, USA), and the cDNA samples were stored at − 80 °C until further analysis.

Quantitative real-time polymerase chain reaction (qRT-PCR) was conducted using a SYBR Green-based detection system (Applied Biosystems RT-PCR System 2700, USA) to quantify the expression levels of target miRNAs, following the manufacturer’s instructions. Due to the low concentration and short length of circulating miRNAs in serum, conventional RNA integrity assessments such as RIN scores were not applicable. To ensure RNA extraction quality and verify RNA integrity, U6 small nuclear RNA was utilized as the endogenous control across all samples in each qRT-PCR run to normalize miRNA expression levels. Consistent Ct values of U6 among the three studied groups (controls, ESRD-DM, and ESRD-HTN), with no statistically significant differences observed (*p* > 0.05), confirming its stability and suitability as an endogenous control for normalization of miRNA expression in this study. Relative quantification of miRNA expression was calculated using the 2^−ΔΔCT^ method^22^. As follow: (1) the ΔCT value was calculated by subtracting the Ct value of the endogenous control (U6) from the Ct value of the target miRNA (ΔCT = Ct-miRNA – Ct-U6); (2) the ΔΔCT was then determined by subtracting the average ΔCT of the control group (used as the calibrator) from the ΔCT of each test sample (ΔΔCT = ΔCT-sample – ΔCT-control; 3) fold changes in miRNA expression were calculated as 2^−ΔΔCT^. All reactions were performed in duplicate, and the mean Ct values were used for calculations. The specific primer sequences for the target miRNAs and U6 are provided in Table 1.

**Table 1:** The MiRNAs primers utilized in the study.

miRNAs	Sequence of primers 5′ → 3′	References
miR-21 (F)	5′-TAGCTTATCAGACTGATGTTGA-3′	^23﻿^
miR-21 (R)	5′-GTGCAGGGTCCGAGGT-3′	
miR-126-3p (F)	5′-TAGCACCATTTGAAATCAGTGTT-3′	^24^
miR-126-3p (R)	5′-GTGCAGGGTCCGAGGT-3′	
miR-192 (F)	5′-CTGACCTATGAATTGACAGCC-3′	^25^
miR-192 (R)	5′-GAGAAGAT TAGCATGGCCC-3′	
U6 (F)	5′-CTCGCTTCGGCAGCACA-3′	^26^
U6 (R)	5′-AACGCTTCACGAATTTGCGT-3′	

### Statistical analysis

The statistical power and sample size were calculated using G*Power (version 3.1). Statistical analyses were conducted using SPSS software (version 27; IBM Corp., USA). Normality of all measured variables was evaluated prior to analysis using the Shapiro–Wilk test^27^. Data were presented as mean ± standard deviation (SD) for continuous variables and as numbers and percentages for categorical variables. Group comparisons were performed using one-way analysis of variance (ANOVA), followed by the least significant difference (LSD) t-test for post hoc pairwise comparisons. Furthermore, the Benjamini-Hochberg false discovery rate (BH-FDR) correction was conducted for the miRNAs multiple evaluations and miRNA with a *q*-value > 0.05 considered statistically significant. Pearson correlation analysis and linear regression were employed to assess the relationships between miRNA expression levels and clinical parameters while, Partial correlation analysis was utilized to determine the association of the studied miRNAs with the clinical variables after adjusting age and sex. To evaluate the diagnostic performance of the target miRNAs, receiver operating characteristic (ROC) curve analysis was performed, and the area under the curve (AUC) was calculated to quantify discriminative accuracy. The optimal cut-off point for each miRNA was selected based on the Youden Index^28^which identifies the threshold that provides the best balance between sensitivity and specificity to identify ESRD patients with hypertension and diabetes. Consequently, a separate cutoff value for each comparison (ESRD-HTN versus healthy controls and ESRD-DM versus healthy controls) was derived from group-wise ROC curves. A two-sided *P*-value of less than 0.05 was considered statistically significant throughout the analyses.

## Results

**Fig. 1:**
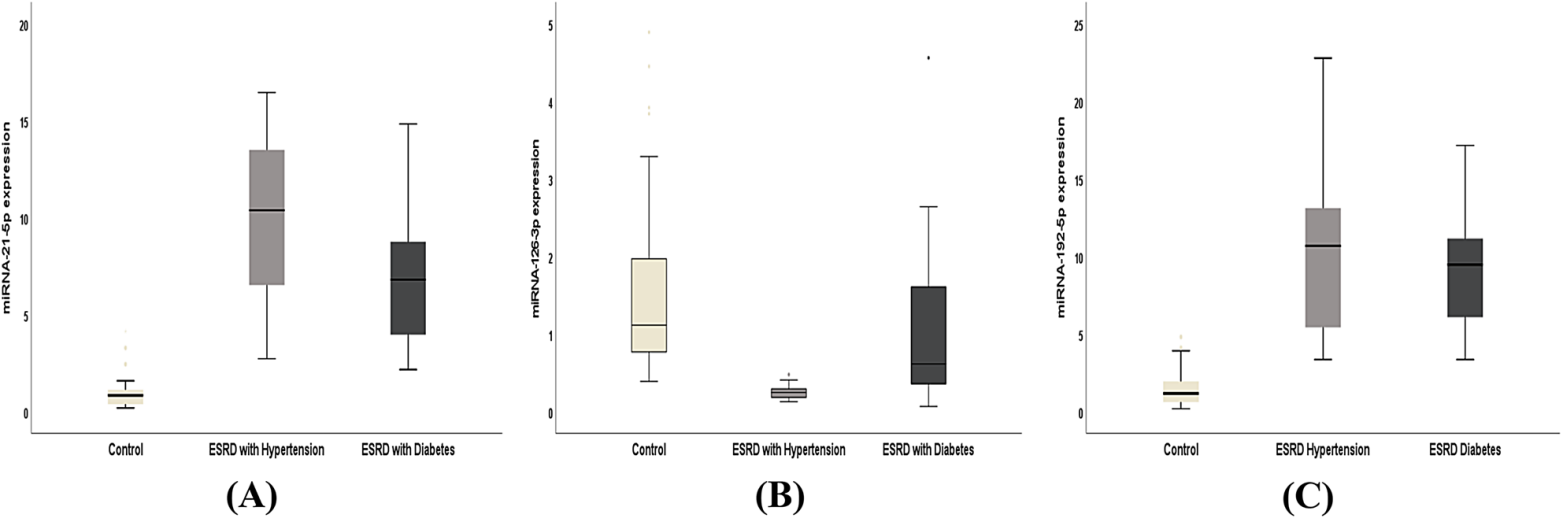
The expression levels of miR-21-5p, miR-126-3p, and miR-192-5p in the studied groups.

**Fig. 2:**
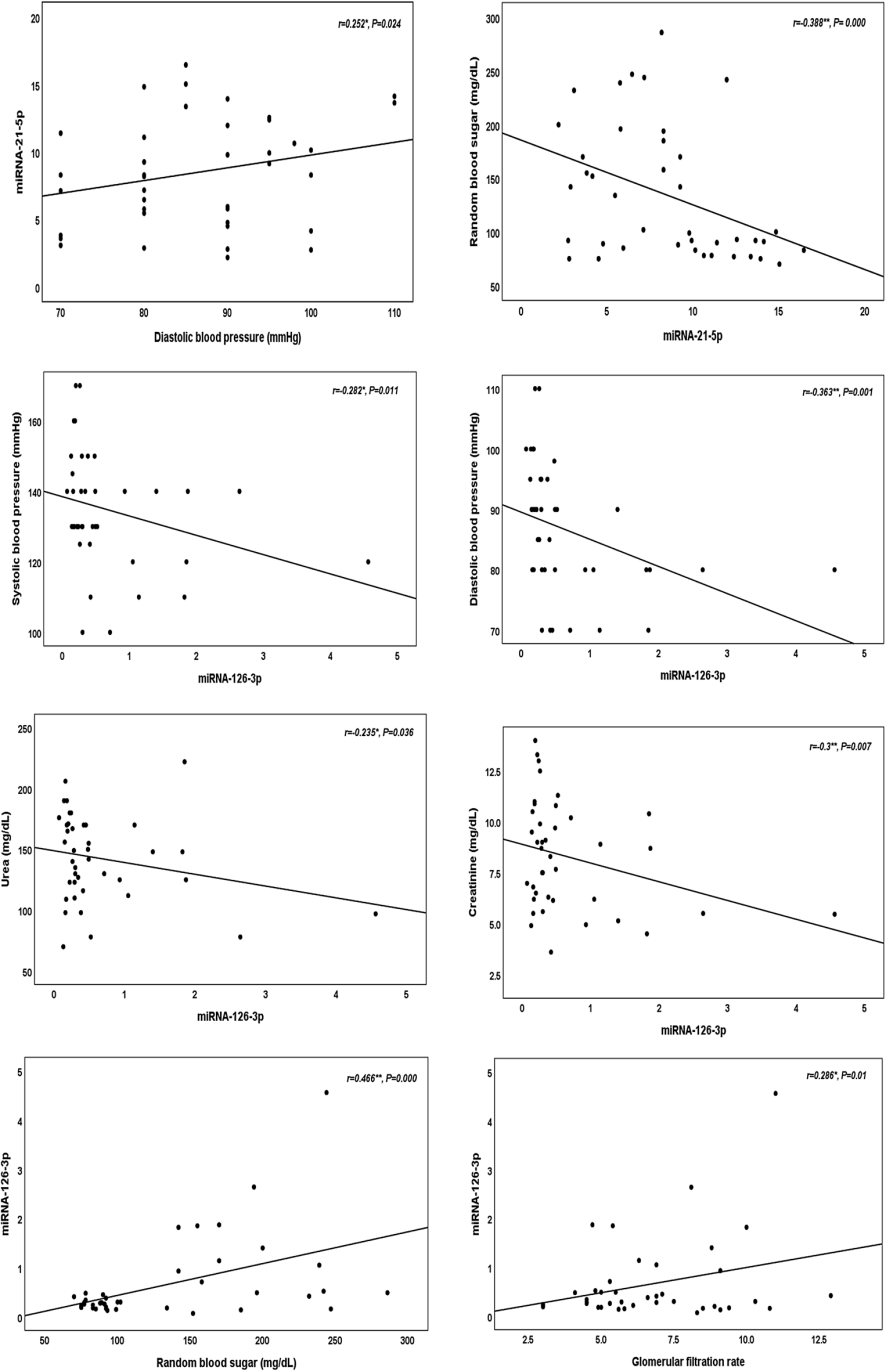
Correlations of miRNAs with the significant parameters in ESRD patients.

**Fig. 3:**
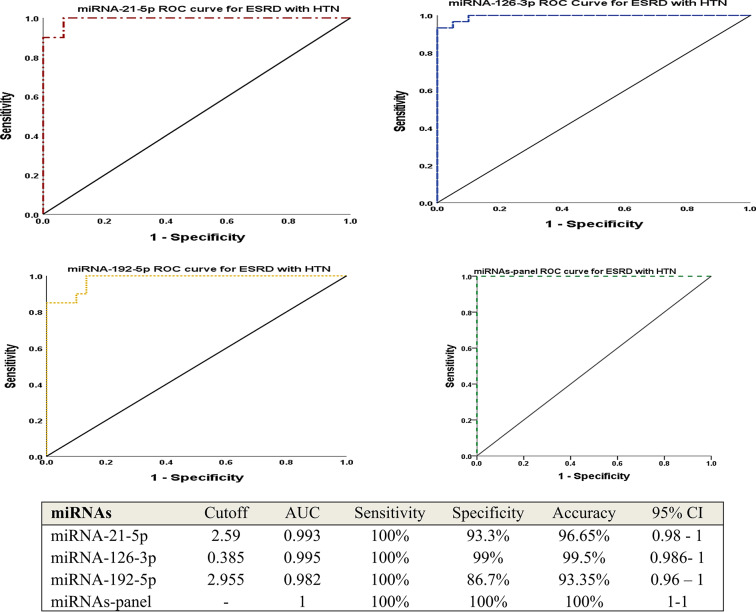
ROC analysis for the discriminatory ability of studied miRNAs to identify ESRD patients with hypertension. miRNA: micro-RNA; ESRD: end stage renal disease; HTN: hypertension; AUC: area under the curve; CI: confidence interval.

**Fig. 4:**
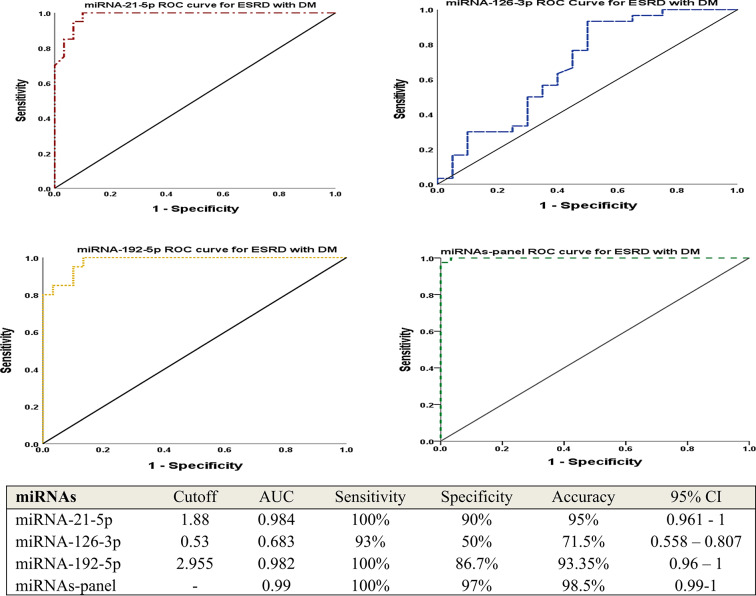
ROC analysis for the discriminatory ability of studied miRNAs to identify ESRD patients with diabetes mellitus. miRNA: micro-RNA; ESRD: end stage renal disease; DM: diabetes mellitus; AUC: area under the curve; CI: confidence interval.

Table 2 revealed the anthropometric and clinical features of the three considered groups. There were significant increases in the levels of random blood sugar (RBS) and HbA1c in the end-stage renal disease with DM group also there was an elevation in the blood pressure among ESRD with HTN group. Glomerular filtration rate, urea, and creatinine were significantly different in the two ESRD groups (with HTN and with DM) compared to the control group (*P* < 0.001). As demonstrated in Table 3; Fig. 1 (A, B, and C), circulating miRNA-126-3p was significantly declined among ESRD groups in comparison to normal controls (*P* < 0.001). While, there were significant increases in miRNA-21-5p and miRNA-192-5p levels between the two ESRD groups relative to normal controls (*P* < 0.001). Besides, there were significant differences concerning the miRNAs (21-5p and 126-3p) expression between the ESRD groups (HTN and DM) however there was no difference regarding miRNA-192-5p (*P* = 0.111). To control for multiple miRNA testing, the Benjamini-Hochberg false discovery rate (FDR) correction was applied to adjust the *p*-values derived from the three studied miRNAs expression analyses. After correction, the expression levels of miRNA-21-5p and miRNA-192-5p in ESRD cases with HTN and DM relative to controls, as well as the expressions of miRNA-126-3p in ESRD-HTN patients compared to controls, remained statistically significant with adjusted *q*-values of 0.006. The second comparison for miRNA-126-3p between ESRD-DM and control groups showed a *q*-value of 0.023, which also met the conventional threshold for significance (*q* < 0.05). Regarding the comparison between the two patients’ groups, both miRNA-21 and miRNA-126 remained statistically significant with adjusted *q*-values of 0.003. In contrast, miRNA-192 did not reach statistical significance with or without correction, with a *q*-value of 0.111. The Pearson correlation analyses of the clinical parameters and the estimated miRNAs in the studied groups were interpreted in Table 4; Figs. 2, there was a positive association between miRNA-21-5p and diastolic blood pressures (DBP) while a negative relation with RBS. For miRNA-126-3p, there were correlations with DBP, RBS, urea, creatinine, GFR, and calcium. Furthermore, after adjusting for age and sex, partial correlation analysis revealed similar associations between miRNA expression levels and clinical variables in ESRD patients, consistent with the findings from Pearson correlation analysis, as shown in Table 5. Tables 6 and 7, and 8 illustrated the multiple linear regression analyses for the associations of the studied miRNAs expression level and different variables among ESRD cases, which shown a significant linking for miRNA-21-5p with gender, DBP, urea, creatinine, GFR, Na^+^, and K^+^. Regarding miRNA-126-3p, there were significant relationship with sex, urea, creatinine, and Ca^++^. Concerning miRNA-192-5p, the association was with hemoglobin and GFR. The ROC curve analyses were conducted to assess the AUC, sensitivity, specificity, and accuracy for the tested miRNAs (21-5p, 126-3p, and 192-5p) in the ESRD with HTN and ESRD with DM groups versus to the control group, as signified in Figs. 3 and 4, to discriminate the competence of these miRNAs as biomarkers for ESRD and its related hypertension and diabetes mellitus. The accuracies of ROC analyses for miR-21-5p, miR-126-3p, and miR-192-5p among ESRD with HTN were 96.65%, 99.5%, and 93.35%; respectively and in ESRD with DM were 95%, 71.5%, and 93%. Thus, the ROC for the miRNA-126-3p in ESRD patients with HTN revealed the uppermost diagnostic accuracy (99.5%). Additionally, regarding the panel miRNAs, the miRNAs-panel for ESRD patients with HTN had the highest AUC = 1 and accuracy = 100% as demonstrated in Fig. 3. While, for ESRD cases with DM had AUC = 0.99 and accuracy = 98.5% as represented in Fig. 4.

**Table 2:** Descriptive and clinical data for studied groups.

Variables	Control (*n* = 30)	ESRD with hypertension (*n* = 40)	ESRD with diabetes mellitus (*n* = 40)	*P* values
Age (years)	52.4 ± 8	53.2 ± 9.7	53.9 ± 4.4	0.721
Sex M/F (n %)	16 (53.3%)/ 14 (46.7%)	22 (55%)/ 18 (45%)	20 (50%)/ 20 (50%)	0.902
Systolic blood pressure (mmHg)	120	143	127	**< 0.001**
Diastolic blood pressure (mmHg)	80	92	81	**< 0.001**
Hemoglobin (mg/dL)	13.1 ± 0.68	9.36 ± 1.3	8.75 ± 2.7	**< 0.001**
Random blood sugar (mg/dL)	93.5 ± 9.2	84.7 ± 8.8	183.9 ± 51.65	**< 0.001**
Glycated hemoglobin %	4.56 ± 0.34	---	7.24 ± 0.87	**< 0.001**
Serum urea (mg/dL)	23.37 ± 3.66	136.6 ± 30.7	148.35 ± 39.2	**< 0.001**
Serum creatinine (mg/dL)	0.768 ± 0.086	9.12 ± 2.6	7.45 ± 2.4	**< 0.001**
Glomerular filtration rate (mL/min/1.73m^2^)	93.66 ± 5.09	6.03 ± 2.22	7.43 ± 2.35	**< 0.001**
Sodium (mEq/L)	141.3 ± 4.15	137.4 ± 4.16	134.9 ± 3.3	**< 0.001**
Potassium (mEq/L)	4.26 ± 0.41	5.22 ± 0.5	5.06 ± 0.67	**< 0.001**
Calcium (mmol/L)	1.19 ± 0.046	1.15 ± 0.076	1.16 ± 0.084	0.065

Data represented as mean ± standard deviation (SD) or as number %

*P* < 0.01 is highly significant.

**Table 3:** The expression levels of miRNA-21-5p, miRNA-126-3p, and miRNA-192-5p in the studied groups (***p***-values were adjusted for multiple testing using the Benjamini–Hochberg method to calculate *q*-values).

	Mean	Standard deviation		95% confidence interval	*P* values		P* values	q-values		q*-values
**miRNA-21-5p**										
Control	0.981	0.9051		0.643–1.319	Reference					
ESRD with HTN	9.998	4.0875		8.691–11.305	**< 0.001**		< 0.001	**0.006**		0.003
ESRD with DM	6.9965	3.3068		5.939–8.054	**< 0.001**			**0.006**		
**miRNA-126-3p**										
Control	1.5727	1.2494		1.106–2.039	Reference					
ESRD with HTN	0.2575	0.0909		0.228–0.287	**< 0.001**		< 0.001	**0.006**		0.003
ESRD with DM	1.0595	1.076		0.715–1.404	**0.023**			**0.023**		
**miRNA-192-5p**										
Control	1.5373	1.1965		1.091–1.984	Reference					
ESRD with HTN	10.6155	6.099		8.665–12.566	**< 0.001**		0.111	**0.006**		0.111
ESRD with DM	9.05	3.721		7.86–10.24	**< 0.001**			**0.006**		

Control group: normal healthy

ESRD with HTN group: end stage renal disease with hypertension

ESRD with DM group: end stage renal disease with diabetes mellitus

*p* and p* values represent the unadjusted significance

q and q* values represent the adjusted p-values to account for multiple miRNAs comparisons using the Benjamini-Hochberg correction

p and q values: ESRD vs. Control; p* and q* values: ESRD HTN vs. ESRD DM

*p and q < 0.05 are significant p and q < 0.01 are highly significant*

**Table 4:** Pearson correlation between the examined MiRNAs expression levels and different parameters in ESRD patients (unadjusted sex and age).

Parameter	miRNA-21-5p		miRNA-126-3p		miRNA-192-5p	
	*r*	*p*	*r*	*p*	*r*	*p*
Systolic blood pressure (mmHg)	0.181	0.108	−0.282*	**0.011**	0.020	0.858
Diastolic blood pressure (mmHg)	0.252*	**0.024**	−0.363**	**0.001**	0.099	0.381
Hemoglobin (mg/dL)	−0.064	0.573	0.081	0.475	0.009	0.384
Random blood sugar (mg/dL)	−0.388**	**0.000**	0.466**	**0.000**	−0.172	0.126
Urea (mg/dL)	−0.196	0.081	−0.235*	**0.036**	−0.035	0.755
Creatinine (mg/dL)	0.147	0.192	−0.3**	**0.007**	−0.084	0.459
Glomerular filtration rate (mL/min/1.73 m^2^)	−0.193	0.087	0.286*	**0.010**	0.021	0.854
Sodium (mEq/L)	0.140	0.216	−0.011	0.921	−0.107	0.343
Potassium (mEq/L)	−0.089	0.435	−0.206	0.067	−0.208	0.064
Calcium (mmol/L)	−0.100	0.379	0.311**	0.005	**0.033**	0.773

Pearson correlation was applied without age and sex adjustment

*r*: correlation coefficient *p*: significant value

*P < 0.05 is significant **P < 0.01 is highly significant

**Table 5:** Partial correlation for the assessed MiRNAs expression levels with different variables in ESRD cases (adjusted age and sex).

Parameter	miRNA-21-5p		miRNA-126-3p		miRNA-192-5p	
	*r*	*p*	*r*	*p*	*r*	*p*
Systolic blood pressure (mmHg)	0.204	0.073	−0.293*	**0.009**	0.033	0.775
Diastolic blood pressure (mmHg)	0.256*	**0.023**	−0.355**	**0.001**	0.153	0.181
Hemoglobin (mg/dL)	−0.082	0.477	0.112	0.330	0.178	0.120
Random blood sugar (mg/dL)	−0.397**	**0.000**	0.469**	0.000	−0.185	0.104
Urea (mg/dL)	−0.194	0.089	−0.225*	0.048	−0.033	0.775
Creatinine (mg/dL)	0.189	0.097	−0.308**	0.006	−0.014	0.903
Glomerular filtration rate (mL/min/1.73 m^2^)	−0.212	0.062	0.292*	0.009	0.005	0.962
Sodium (mEq/L)	0.155	0.175	−0.017	0.882	−0.120	0.296
Potassium (mEq/L)	−0.105	0.361	−0.184	0.098	−0.130	0.258
Calcium (mmol/L)	−0.125	0.277	0.319**	**0.004**	0.002	0.988

Partial correlation analysis was performed with adjusting age and sex

*r*: correlation coefficient; *p*: significant value; *P < 0.05 is significant; **P < 0.01 is highly significant

**Table 6:** Multiple linear regression analysis for the association between miRNA-21-5p expression levels and some studied parameters in ESRD patients.

Parameters	Unstandardized coefficients		Standardized coefficients	t	Significant	95% Confidence interval for β	
	β	SE	Beta			Lower bound	Upper bound
Age	-0.161	0.115	-0.216	-1.407	0.171	-0.397	0.074
Sex	9.022	1.663	1.382	5.426	**0.000**	5.605	12.440
Systolic blood pressure (SBP)	-0.031	0.055	-0.114	-0.570	0.574	-0.145	0.082
Diastolic blood pressure (DBP)	0.154	0.059	0.419	2.623	**0.014**	0.033	0.274
Hemoglobin (Hb)	0.15	0.156	0.122	0.965	0.344	-0.170	0.470
Random blood sugar (RBS)	-0.037	0.019	-0.580	-1.954	0.062	-0.076	0.002
Glycated hemoglobin (HbA1c)	0.397	1.194	0.104	0.332	0.743	-2.059	2.852
Urea	-0.029	0.01	-0.344	-2.969	**0.006**	-0.049	-0.009
Creatinine	2.481	0.692	1.801	3.585	**0.001**	1.059	3.904
Glomerular filtration rate (GFR)	1.462	0.549	1.041	2.663	**0.013**	0.334	2.591
Sodium (Na^+^)	-0.844	0.155	-0.842	-5.432	**0.000**	-1.164	-0.525
Potassium (K^+^)	-2.148	0.615	-0.433	-3.495	**0.002**	-3.412	-0.885
Calcium (Ca^++^)	4.604	6.173	0.117	0.746	0.462	-8.085	17.294
β: beta-coefficient, SE: standard error							

*P*<0.05 is significant, *P*<0.01 is highly significant

**Table 7:** Multiple linear regression analysis for the association between miRNA-126-3p expression levels and some parameters in ESRD patients.

Parameters	Unstandardized coefficients		Standardized coefficients	t	Significant	95% Confidence interval for β	
	β	SE	Beta			Lower bound	Upper bound
Age	−0.161	0.115	−0.216	−1.407	0.171	−0.397	0.074
Sex	9.022	1.663	1.382	5.426	**0.000**	5.605	12.440
Systolic blood pressure (SBP)	−0.031	0.055	−0.114	−0.570	0.574	−0.145	0.082
Diastolic blood pressure (DBP)	0.154	0.059	0.419	2.623	**0.014**	0.033	0.274
Hemoglobin (Hb)	0.15	0.156	0.122	0.965	0.344	−0.170	0.470
Random blood sugar (RBS)	−0.037	0.019	−0.580	−1.954	0.062	−0.076	0.002
Glycated hemoglobin (HbA1c)	0.397	1.194	0.104	0.332	0.743	−2.059	2.852
Urea	−0.029	0.01	−0.344	−2.969	**0.006**	−0.049	−0.009
Creatinine	2.481	0.692	1.801	3.585	**0.001**	1.059	3.904
Glomerular filtration rate (GFR)	1.462	0.549	1.041	2.663	**0.013**	0.334	2.591
Sodium (Na^+^)	−0.844	0.155	−0.842	−5.432	**0.000**	−1.164	−0.525
Potassium (K^+^)	−2.148	0.615	−0.433	−3.495	**0.002**	−3.412	−0.885
Calcium (Ca^++^)	4.604	6.173	0.117	0.746	0.462	−8.085	17.294

β: beta-coefficient, SE: standard error

*P* < 0.05 is significant, *P* < 0.01 is highly significant

**Table 8:** Multiple linear regression analysis for the association between miRNA-192-5p expression levels and some variables in ESRD patients.

Parameters	Unstandardized coefficients		Standardized coefficients	t	Significant	95% Confidence interval for β	
	β	SE	Beta			Lower bound	Upper bound
Age	−0.046	0.045	−0.191	−1.023	0.316	−0.139	0.047
Sex	−1.71	0.657	−0.805	−2.602	**0.015**	−3.061	−0.359
Systolic blood pressure	0.025	0.022	0.282	1.159	0.257	−0.02	0.07
Diastolic blood pressure	−0.041	0.023	−0.344	−1.775	0.088	−0.089	0.007
Hemoglobin	0.077	0.061	0.192	1.251	0.222	−0.049	0.203
Random blood sugar	0.007	0.008	0.341	0.946	0.353	−0.008	0.023
Glycated hemoglobin	−0.269	0.472	−0.217	−0.570	0.573	−1.239	0.701
Urea	−0.012	0.004	−0.433	−3.078	**0.005**	−0.02	−0.004
Creatinine	−0.604	0.274	−1.347	−2.208	**0.036**	−1.166	−0.042
Glomerular filtration rate	−0.214	0.217	−0.469	−0.987	0.333	−0.66	0.232
Sodium	0.033	0.061	0.1	0.533	0.598	−0.094	0.159
Potassium	0.293	0.243	0.181	1.205	0.239	−0.207	0.792
Calcium	5.19	2.44	0.406	2.127	**0.043**	0.175	10.205

β: beta-coefficient, SE: standard error

*P* < 0.05 is significant, *P* < 0.01 is highly significant

## Discussion

Circulating miRNAs have emerged as promising diagnostic biomarkers due to their stability in bodily fluids and the availability of reliable detection methods. These small non-coding RNAs play critical roles in regulating key biological processes such as apoptosis, cellular proliferation, and development, thereby influencing disease progression^29,30^. In the context of chronic kidney disease, accumulating evidence suggests that certain miRNAs are differentially expressed based on disease severity, making them potential markers for CKD diagnosis and monitoring^31,32^. However, data on circulating miRNAs in end-stage renal disease, particularly in relation to miRNA-21-5p, miRNA-126-3p, and miRNA-192-5p, remain limited. Thus, the current study aimed to evaluate the serum expression levels of these specific miRNAs in ESRD patients to elucidate their potential roles in ESRD pathogenesis and to assess their prognostic utility.

The present study demonstrated a significant decrease in circulating miRNA-126-3p levels among ESRD patients with both hypertension and diabetes mellitus compared to healthy controls. Notably, miR-126-3p expression was also significantly lower in the HTN group than in the DM group. These findings align with previous reports indicating that miR-126 expression is reduced in ESRD and hemodialysis patients^1,32,33^. In a study examining CKD patients, miR-126 levels were substantially lower in ESRD subjects compared to both healthy controls and those with earlier stages of CKD, with fold changes of 352 and 358, respectively^34^. Another study identified a marked reduction in miR-126 levels in CKD, where individuals in the highest tertile of miR-126 expression exhibited a lower risk of CKD compared to those in the lowest tertile^35^. MiR-126-3p was reported to be down-regulated in hypertension-associated chronic kidney disease and in diabetic kidney disease^18^. It was exposed that serum and whole blood samples of miRNA-126 were lower in the general population and in those with comorbidities such as DM and HTN. This was positively correlated with glomerular filtration rate and inversely correlated with the prevalence of CKD^36^. This inverse relationship was evident in our study, wherein lower serum miR-126-3p expression was associated with more advanced renal dysfunction and reduced GFR.

Beyond its role in renal pathology, miR-126-3p is a well-established regulator of vascular endothelial function. It has been implicated in the modulation of vascular smooth muscle cell turnover, angiogenesis, and endothelial repair^37,38^. Reduced miR-126 expression could, therefore, contribute to vascular abnormalities commonly observed in CKD patients, potentially exacerbating atherosclerosis and vascular dysfunction. Despite these findings, some studies have reported elevated miR-126 levels in the plasma, urine, and serum of diabetic kidney disease (DKD) patients, possibly indicating a compensatory response to endothelial stress^18^. However, the relationship between miRNA levels and renal function remains inconsistent across studies. Variability in miRNA expression may be influenced by factors such as renal clearance, RNase aggregation, and miRNA binding to protective carriers like exosomes and Argonaute proteins^34^. Further research is needed to clarify these mechanisms and to explore the potential of miR-126 as a biomarker for both renal and vascular pathologies in CKD patients.

Regarding miRNA-21-5p, this study found a significant elevation in miRNA-21-5p levels among both ESRD groups (HTN and DM) compared to the control group. Consistent with our data some studies reports demonstrating upregulation of miR-21 in urine and urine exosomes of CKD patients^39,40^. Similarly, other studies have consistently reported increased miR-21 expression in DKD, suggesting its potential role as a biomarker for disease progression^18,41–43^. Whilst, conflicting evidence exists, with some studies identifying miR-21 downregulation in serum samples^39^ and others detecting no significant changes in urine or plasma miR-21 levels between cases and controls^44^. The observed overexpression of miR-21 in ESRD patients may be attributed to its involvement in key biological processes, including cellular proliferation, differentiation, and apoptosis. Notably, miR-21 is a well-established regulator of kidney fibrosis, mediating epithelial injury responses and promoting fibrotic pathways through metabolic reprogramming^45,46^. Sun et al. identified miR-21 as a central factor in fibroblast activation in progressive diseases, further highlighting its role in fibrosis development^12^. In rodent models of kidney disease, Zhong et al. demonstrated that miR-21 inhibition mitigated renal fibrosis, indicating its potential as a therapeutic target^47^. Similarly, Gomez et al. indicated a protective effect against fibrosis in miR-21-deficient mice^48^. The fibrogenic effects of miR-21 are partly mediated through its influence on lipid metabolism and oxidative stress pathways. Overexpression of miR-21 has been associated with enhanced reactive oxygen species (ROS) production and mitochondrial dysfunction, further exacerbating tissue fibrosis^49,50^. Conversely, silencing of miR-21 has been shown to restore mitochondrial function, reduce inflammation, and decrease fibrogenesis in renal tubular and glomerular cells^45^. These findings suggest that targeting miR-21 may offer therapeutic potential in mitigating CKD-related fibrosis and renal damage.

Concerning miRNA-192-5p, the present study identified a significant elevation in circulating miR-192-5p levels in ESRD patients relative to healthy controls. In the same line with our data, some previous studies have observed an increase in the expressions of miR-192 in individuals with diabetic kidney disease compared with controls^51,52^. Jia et al. further informed that miR-192 expression is elevated in the early stages of diabetic nephropathy (DN), correlating positively with albuminuria, thereby suggesting a potential profibrotic role for this miRNA^53^. Nonetheless, the role of miR-192 in renal disease appears to be complex and context-dependent. Jenkins et al. highlighted that miR-192 exerts pleiotropic effects in the kidney, with its function as either a profibrotic or antifibrotic factor varying across different cell types. Supporting this dual role, miR-192 has been shown to have a protective effect against kidney fibrosis^54^. Conversely, other reports have documented reduced miR-192 expression in DN patients, associating lower levels with decreased renal function and increased kidney damage^55–57^. This apparent discrepancy suggests that miR-192 expression may fluctuate throughout disease progression, potentially rising in the initial stages of renal injury but declining as fibrosis and albuminuria worsen. Consequently, miR-192 may serve as a biomarker for early-stage renal disease, with its reduced expression potentially indicating advancing renal dysfunction^18^.

While the individual miRNAs assessed in this study demonstrated promising diagnostic performance, the use of single biomarkers in isolation presents inherent limitations. miRNA expression can be influenced by various factors such as inflammation, comorbid conditions, medication use, and individual biological variability. This may affect the reliability and reproducibility of single-miRNA-based diagnostics across diverse populations^58^. A combined miRNA panel or multi-marker approach could enhance diagnostic accuracy by capturing a broader spectrum of disease-related molecular changes and reducing the impact of individual variability. Such panels have been shown in other disease contexts to improve sensitivity, specificity, and overall predictive value^59,60^. In the context of chronic kidney disease, integrating multiple miRNAs, potentially alongside existing clinical markers, could strengthen risk prediction and early detection strategies compared to single-marker approaches. Future studies with more comprehensive biomarker analyses are warranted to explore and validate the utility of such combined diagnostic models^61^. As we previously discussed, the circulating levels of miR-126-3p, miR-21-5p, and miR-192-5p were significantly altered in ESRD patients with concomitant hypertension and diabetes mellitus. These findings were further supported by receiver operating characteristic (ROC) analysis, which demonstrated high diagnostic accuracy for each miRNA, underscoring their potential utility as biomarkers for ESRD detection and prognosis. Furthermore, panels of the examined miRNAs, such as miR-21-5p, −126-3p, and − 192-5p, had better overall AUCs for discriminating ESRD patients with DM and HTN. Thus, utilizing miRNA panels as potential markers for ESRD is more beneficial than depending on a particular miRNA. Notably, the robust performance of these miRNAs in differentiating ESRD patients from controls suggests their relevance in identifying individuals at increased risk of renal dysfunction associated with metabolic comorbidities.

To date, there is limited evidence that medications commonly used in patients with ESRD, such as antihypertensive or antidiabetic agents, directly influence circulating levels of miRNA‑21‑5p, miRNA‑126‑3p, or miRNA‑192‑5p. A cross-sectional study reported that antihypertensive agents may alter miRNA expression profiles in individuals with hypertension, but effects on specifically miRNA‑126‑3p were not confirmed in that cohort^62^. Some studies have focused on miRNA‑126 in the context of cardiovascular (CV) health and vascular remodeling, but none have definitively linked standard antihypertensive or anti‑diabetic agents to changes in circulating miRNA‑126‑3p, miRNA‑21‑5p, or miRNA‑192‑5p^63^. Moreover, the effects of CV drugs on various circulating miRNAs are often inconsistent and context-dependent^64,65^. A recent review examined the role of miR-126 in clinically significant diseases and the future potential of microRNA-based therapies. Nevertheless, successful clinical application of this therapy requires further research to address various treatment-related issues^66^. In our patient groups, no participants were on medications known to directly alter the studied miRNAs. Additionally, we performed the multiple-miRNAs corrections via the Benjamini-Hochberg method, which revealed significant statistical significance as the ANOVA test without corrections. Thus, the medication regimen of our patients could not significantly compromise the validity of our results. However, we acknowledge that the possibility of drug-induced modulation cannot be entirely ruled out, empirical data remains sparse, and further focused studies are needed.

The practical application of miRNA-based testing in low-resource settings presents several challenges. Currently, miRNA quantification typically requires specialized equipment such as real-time PCR machines and trained personnel, which may limit accessibility in regions with constrained healthcare infrastructure^67^. Additionally, the cost of reagents and standardized protocols for circulating miRNA analysis remains relatively high. However, ongoing advances in point-of-care molecular diagnostics, including the development of simplified, low-cost miRNA detection platforms, hold promise for expanding access to biomarker-based screening^68^. The integration of miRNA testing into existing public health frameworks could eventually enhance early detection of ESRD in underserved populations, provided that future research focuses on cost-effectiveness, assay simplification, and health system integration.

### Limitations

Although the findings of this study provide valuable insights into the potential utility of miRNAs as biomarkers for ESRD using ROC analysis, the study has several limitations that should be acknowledged. First, the relatively small sample size of 110 participants across three groups represents a key limitation. To confirm the preliminary findings and better assess the clinical relevance of the identified biomarkers, it is essential that future research involve larger, multicenter populations with more comprehensive data collection. Second, subgroup analyses were not performed based on specific medication use; their potential influence on miRNA expression was not evaluated. Future prospective studies with larger cohorts and detailed stratification based on treatment types are needed to further clarify the impact of medication use on the target miRNA expressions. Finally, the absence of cross-validation or the use of an independent validation cohort to confirm the diagnostic performance of the identified miRNAs. Therefore, larger, independent cohorts and cross-validation approaches are warranted in future studies to validate these findings and ensure their robustness and generalizability.

## Conclusions

Circulating miRNAs present in biofluids have emerged as promising candidates for non-invasive biomarkers, offering potential diagnostic and prognostic utility for various diseases, including ESRD. However, the precise role of specific miRNAs in ESRD pathogenesis and their regulatory mechanisms remain incompletely understood. The present study underscores the diagnostic relevance of miR-21, miR-126-3p, and miR-192 as potential biomarkers for ESRD, independent of comorbid conditions such as diabetes and hypertension. These findings support further exploration into the clinical application of these miRNAs as accessible indicators of renal dysfunction. To advance the clinical translation of miRNA biomarkers, future studies should focus on elucidating the molecular targets and signaling pathways influenced by these miRNAs, particularly in the context of renal fibrosis, inflammation, and oxidative stress. Investigating the gene networks regulated by miR-21, miR-126-3p, and miR-192 could provide deeper insights into their roles in kidney disease progression and reveal potential therapeutic targets. Additionally, the absence of cross-validation and external validation limits the generalizability of these results. Therefore, prospective studies involving larger and more diverse patient cohorts, as well as independent validation, are warranted to validate these findings and assess the prognostic accuracy of these miRNAs in predicting ESRD onset and progression.

## Data Availability

The data used to support the findings of this study are available within the article.
